# *E. coli*-Produced Monophosphoryl Lipid a Significantly Enhances Protective Immunity of Pandemic H1N1 Vaccine

**DOI:** 10.3390/vaccines8020306

**Published:** 2020-06-16

**Authors:** Quyen Thi Nguyen, Eunjin Kim, Jihyun Yang, Chankyu Lee, Da Hui Ha, Choon Geun Lee, Ye Ram Lee, Haryoung Poo

**Affiliations:** 1 Infectious Disease Research Center, Korea Research Institute of Bioscience and Biotechnology (KRIBB), Daejeon 34141, Korea; quyenbio@kribb.re.kr (Q.T.N.); ejkim@kribb.re.kr (E.K.); jhyang@kribb.re.kr (J.Y.); 2 Department of Biosystems and Bioengineering, KRIBB School of Biotechnology, University of Science and Technology, Daejeon 34113, Korea; 3 College of Veterinary Medicine, Chungnam National University, Daejeon 34134, Korea; 4 Eubiologics. Co., Ltd., V Plant, Gangwon-do 24410, Korea; cklee@eubiologics.com (C.L.); hdh1216@eubiologics.com (D.H.H.); lck6919@eubiologics.com (C.G.L.); yeramme@eubiologics.com (Y.R.L.)

**Keywords:** adjuvant, vaccine efficacy, influenza virus, monophosphoryl lipid A, antibody production, cytotoxic T lymphocyte

## Abstract

Emerging influenza viruses pose an extreme global risk to human health, resulting in an urgent need for effective vaccination against influenza infection. Adjuvants are vital components that can improve vaccine efficacy, yet only a few adjuvants have been licensed in human vaccines. Here, we investigate the adjuvant effects of *Escherichia coli*-produced monophosphoryl lipid A (MPL), named EcML, in enhancing the immunogenicity and efficacy of an influenza vaccine. Similar to MPL, EcML activated dendritic cells and enhanced the antigen processing of cells in vitro. Using ovalbumin (OVA) as a model antigen, EcML increased OVA-specific antibody production, cytotoxic T lymphocyte activity. The safety of EcML was demonstrated as being similar to that of MPL by showing not significant in vitro cell cytotoxicity but transient systemic inflammatory responses within 24 h in OVA immunized mice. Importantly, mice vaccinated with pandemic H1N1 (pH1N1) vaccine antigen, combined with EcML, were fully protected from pH1N1 virus infection by enhanced influenza-specific antibody titers, hemagglutination inhibition titers, and IFN-γ- secreting cells. Taken together, our results strongly suggest that EcML might be a promising vaccine adjuvant for preventing influenza virus infection.

## 1. Introduction

The influenza virus poses a serious global threat to human health and can cause pandemics associated with high mortality worldwide [1]. While influenza vaccines have been used in many countries, the seasonal influenza epidemics have not been well controlled. In addition, the outbreak of the swine-origin H1N1 influenza in 2009 resulted in an estimated 300,000 deaths [2,3], explicitly demonstrating how the influenza vaccination was insufficient for controlling a potential pandemic. Consequently, there is an urgent need to develop an approach to effectively control influenza. One approach to improve the effectiveness of the influenza vaccine is to include adjuvants. While adjuvants have been used in human vaccines to enhance vaccine efficacy for almost a century, only a few adjuvants are licensed. In particular, there are three main adjuvants, alum, MF59, and AS03, which are included in currently licensed influenza vaccines [4]. Despite the fact that alum induces humoral immunity, it weakly stimulates cellular immunity [5]. MF59 has been shown to cause adverse effects, including pain at injection sites and the induction of inflammatory arthritis [6]. Moreover, cases of rare sleeping disorders and narcolepsy after vaccination with AS03-adjuvanted vaccines were reported [7,8]. Therefore, the development of an ideal adjuvant, with regard to potential safety and the ability to enhance both humoral and cellular immune responses that are specific to vaccine antigens, is needed.

Adjuvants targeting pattern-recognition receptors, such as Toll-like receptors (TLRs), are part of a broad approach to stimulate innate immune responses, thereby enhancing antigen-specific immunity and subsequently improving vaccine efficacy [9]. Recently, numerous studies have shown the effects of TLR4-dependent adjuvants on improving vaccine efficacy [10,11,12,13,14]. A TLR4 agonist absorbed on alum in AS04 adjuvant was successfully used in licensed vaccines for human papillomavirus [15] and the hepatitis B virus [16]. Thus, developing TLR4 ligands as adjuvants could be beneficial to improving antiviral vaccines such as influenza vaccine. Monophosphoryl lipid A (MPL) is a detoxified derivative of the lipopolysaccharide (LPS) isolated from *Salmonella minnesota* R595. MPL has been demonstrated to stimulate innate immunity via TLR4 [17] and promote Th1-biased immune responses [18]. Even though MPL has been widely used as an adjuvant in several human vaccines [19], the complicated purification process and prohibitive cost of MPL have precluded its use as an easy-to-use and cost-effective adjuvant. To address these limitations, *Escherichia coli*-produced MPL (named EcML) has been previously produced by direct extraction from genetically engineered *E. coli* [20].

In this study, we first investigated the action mechanisms of EcML to increase innate immunity and tested its adjuvanticity using the model antigen ovalbumin (OVA). EcML triggered the activation of bone marrow-derived dendritic cells (BMDCs) via TLR4 and increased the antigen processing of cells. Compared to OVA alone, OVA plus EcML-immunized mice showed an enhancement in OVA-specific humoral and cellular immune responses. Finally, we explored the adjuvanticity of EcML in enhancing the pandemic H1N1 (pH1N1, A/California/04/09) influenza vaccine efficacy. EcML improved the protective efficacy of the pH1N1 vaccine antigen in mice with 100% survival after viral challenge by increasing influenza-specific antibody (Ab) titers, hemagglutination inhibition (HI) titers, and cytotoxic T lymphocyte (CTL) activity. Collectively, our results strongly suggest that EcML might be a promising adjuvant candidate for influenza vaccines.

## 2. Materials and Methods

### 2.1. Mice and Cells

Six-week-old female C57BL/6 mice were purchased from Orient Bio (Gyeonggi-do, Korea). Six-week-old female wild-type C3H/HeN and congenic TLR4-defective C3H/HeJ mice were purchased from Central Lab. Animal Inc. (Seoul, Korea). All animals were housed in a specific pathogen-free (SPF) facility at the Korea Research Institute of Bioscience and Biotechnology (KRIBB). All experiments employing mice were reviewed and approved by the Institutional Animal Care and Use Committee of the KRIBB. Immature BMDCs were generated using Roswell Park Memorial Institute 1640 media (Gibco) supplemented with 10% heat-inactivated fetal bovine serum (Gibco), 100 U/mL penicillin, 100 mg/mL streptomycin (Gibco), 20 ng/mL murine granulocyte-macrophage colony-stimulating factor (GM-CSF; Peprotech), and 10 ng/mL murine IL-4 (Peprotech) for 7 days followed by changing with fresh media every 3 days, as previously described [21,22].

### 2.2. Preparation of Adjuvants

EcML was produced by Eubiologics. Co., Ltd. (Gangwon-do, Korea) as described previously [20] with slight modifications. Briefly, the EcML was purified from an engineered *E. coli* KHSC0055 and formulated as aqueous formulations using 1,2-dipalmitoyl-sn-glycero-3-phosphocholine and chloroform, and was then evaporated to remove chloroform. The dried EcML was rehydrated in ultrapure water at 0.45 mg/mL, and suspended by sonication at 60 °C. The MPL from *S. minnesota* R595 was purchased from InvivoGen (San Diego, CA, USA) and aqueously formulated using the same processes as for EcML. The resulting EcML and MPL were stored at 4 °C for further use. Alum (Alhydrogel adjuvant 2%) was obtained from InvivoGen.

### 2.3. Preparation of Influenza Virus

The pH1N1 influenza virus was grown in 9- to 10-day-old SPF embryonated chicken eggs (NamDuck SPF, Sungnam, Korea) for 48 h at 37 °C. The viruses were harvested from the allantoic fluids of the eggs by centrifugation at 3500× *g* for 10 min at 4 °C, filtrated through 0.45 μm pore size membrane filters (Merck KGaA, Darmstadt, Germany), and then stored at −80 °C for further use. All viral experiments were implemented under conditions of biosafety level 2.

### 2.4. Cell Viability Assay

Immature BMDCs were stimulated with PBS as a negative control, 0.625, 1.25, 2.5, and 5 μg/mL EcML, 1 μg/mL LPS or 0.5% Triton X-100 (as a positive control) for 24 h at 37 °C. Cell viability was measured using Trypan blue stain 0.4% with the Countess ^TM^ automated cell counter (Thermo Fisher Scientific, Waltham, MA, USA).

### 2.5. In Vitro Activation and Antigen Processing of BMDCs

To investigate the BMDC activation, the cells were stimulated with PBS as a negative control, 0.625, 1.25, 2.5, and 5 μg/mL EcML, or 5 μg/mL MPL for 24 h at 37 °C. The stimulated cells were stained with PE-conjugated monoclonal antibodies (mAbs) against mouse CD40, CD80, CD86, major histocompatibility complex (MHC) class II, and isotype-matched control mAbs (BD Biosciences, San Diego, CA, USA). To examine the antigen processing of BMDCs, we incubated the cells with 5 µg/mL DQ™-OVA, which is OVA conjugated with BODIPY FL dye (a self-quenched dye that emits fluorescence upon proteolytic degradation) (Thermo Fisher Scientific) alone or mixed with either 2.5 μg/mL EcML or MPL for 5 h at 37 °C. The cells were acquired on FACSCalibur flow cytometers (BD Biosciences), and the data were collected and analyzed using FlowJo software version 10 (Tree Star Inc., Ashland, OR, USA).

### 2.6. Western Blotting

Immature BMDCs generated from C3H/HeN and C3H/HeJ mice were serum-deprived for 3 h and then stimulated with EcML at 0.625, 1.25, and 2.5 μg/mL or 2.5 µg/mL MPL for 30 min at 37 °C. The cells were lysed with a lysis buffer containing protease inhibitors (Roche Diagnostics, Indianapolis, IN, USA) plus a phosphatase inhibitor cocktail (Sigma-Aldrich). The cell lysates were collected by centrifugation at 13,000× *g* for 15 min at 4 °C, and concentrations of total proteins were determined using a bicinchoninic acid protein assay (Thermo Fisher Scientific). The cell lysates were separated by 12% SDS-PAGE and transferred to PVDF membranes (Merck KGaA). The blots were blocked with 5% skim milk in Tris-buffered saline containing 0.1% Tween-20 and probed with anti-phospho-IκBα rabbit mAb (Cell Signaling Technology, Beverly, MA, USA) for 16 h at 4 °C, followed by incubation with horseradish peroxidase (HRP)-conjugated anti-rabbit immunoglobulin G (IgG) Ab (Cell Signaling Technology). The blots were then visualized by chemiluminescence using an enhanced chemiluminescent substrate kit (GE Healthcare, Uppsala, Sweden). The membrane was then subsequently stripped and reprobed with anti-β-actin mAb (Cell Signaling Technology) as a loading control.

### 2.7. Immunizations and Viral Challenge

C57BL/6 mice were intramuscularly (i.m.) immunized with 10 µg OVA protein (Sigma-Aldrich) alone or in combination with 0.625, 1.25, 2.5, or 5 µg EcML on days 0, 14, and 28. Spleens and sera were collected 2 weeks after the last immunization. In a separate experiment, the mice were i.m. immunized with 10 µg OVA protein alone or mixed with either 2.5 µg EcML or 2.5 μg MPL. The body temperature of the immunized mice was measured at indicated time points using Thermalert TH-5 (Physitemp Instruments Inc., Clifton, NJ, USA). In addition, sera were collected from the mice. In a separate experiment, the mice were i.m. immunized with 10 µg OVA protein alone or mixed with either 25 µg alum, 2.5 µg EcML, or 2.5 µg MPL on days 0, 14, and 28. Spleens and sera were collected on day 14 after the last immunization. In the influenza experiment, the mice were i.m. immunized with pH1N1 split vaccine antigen (A/California/7/2009 NYMC X-179A H1N1; provided by Mogam Biotechnology Research Institute, Gyeonggi-do, Korea), which contained 0.05 μg hemagglutinin (HA), alone or mixed with 25 μg alum, 2.5 μg EcML, or 2.5 μg MPL on days 0 and 14. Fourteen days after the last immunization, spleens and sera were collected from the vaccinated mice. On day 14 after the final vaccination, the mice were intranasally (i.n.) challenged with a lethal dose (50 LD_50_) of the pH1N1 influenza virus. The body weight and survival of the challenged mice were monitored for 14 days after infection. Mice that lost significantly more than 25% of their body weight were considered to have reached the experimental endpoint and were sacrificed.

### 2.8. HI Assay

HI titers against the pH1N1 influenza virus were determined using sera from the vaccinated mice, as previously described [12]. HI titers were calculated as the reciprocals of the highest dilution of sera at which hemagglutination was completely prevented.

### 2.9. Enzyme-Linked Immunosorbent Assay (ELISA)

Levels of cytokines in the cell culture supernatants were measured using OptEIA kits (BD Biosciences) according to the manufacturer’s instructions. The production of antigen-specific IgG, IgG1, and IgG2b Abs in the sera of the immunized mice were determined by indirect ELISA. Briefly, MaxiSorp 96-well plates (Thermo Fisher Scientific) were coated overnight at 4 °C with 100 µL of 1 µg/mL OVA protein or 0.5 µg/mL pH1N1 split vaccine antigen in carbonate solution, pH 9.5. The plates were blocked with 5% skim milk in PBS for 2 h at 37 °C and were washed with 0.05% Tween-20 in PBS. The plates were then incubated with 100 µL of diluted sera from the vaccinated mice for 2 h at 37 °C, followed by incubation with 100 µL of HRP-conjugated anti-mouse IgG (Cell Signaling Technology), and anti-mouse IgG1 or IgG2b Abs (Southern Biotech, Birmingham, AL, USA) for 1 h at 37 °C. After washing, the reactions were developed with the chromogenic tetramethylbenzidine substrate (BD Biosciences) and then terminated with 2N H_2_SO_4_. The optical density was measured at 450 nm using a Versamax microplate reader (Molecular Devices, San Francisco, CA, USA).

### 2.10. Enzyme-Linked Immunospot (ELISPOT) Assay

The frequencies of antigen-specific IFN-γ-secreting cells were evaluated using a mouse ELISPOT kit (BD Biosciences), as previously described [12,23]. Briefly, 14 days after the last vaccination, splenocytes were obtained from the immunized mice and then plated at 5 × 10^5^ cells/well onto purified anti-IFN-γ-coated ELISPOT plates. The cells were treated with 0.5 µg/well of OVA_257–264_ peptides (Anaspec, San Jose, CA, USA) or the 500 median tissue culture infectious dose (TCID_50_)/well of UV-inactivated pH1N1 influenza virus for 3 days at 37 °C. The spot-forming units (SFUs) of antigen-specific IFN-γ-secreting cells were calculated using an ELISPOT plate reader (Cellular Technology Ltd., Cleveland, OH, USA).

### 2.11. Systemic Inflammatory Responses after Vaccination

To measure the systemic inflammatory responses, sera from the immunized mice were collected. Levels of inflammatory cytokines in the pooled sera (n = 5 per group) were measured using the Lengendplex mouse inflammation panel (13-plex, Biolegend, San Diego, CA, USA) according to the manufacturer’s instructions.

### 2.12. Statistical Analysis

All of the data were presented as the means ± standard deviations (SDs) and represented three independent experiments. Statistically significant differences between the two and multiple groups were assessed using the two-tailed Student’s *t*-test and one-way ANOVA, followed by Bonferroni’s correction (ANOVA/Bonferroni), respectively. The *p* values less than 0.05 (*p* < 0.05) were considered to be statistically significant. All the analyses were implemented using GraphPad Prism software (GraphPad, San Diego, CA, USA).

## 3. Results

### 3.1. EcML Enhances Activation and Antigen Processing of Dendritic Cells (DCs) In Vitro

**Figure 1:**
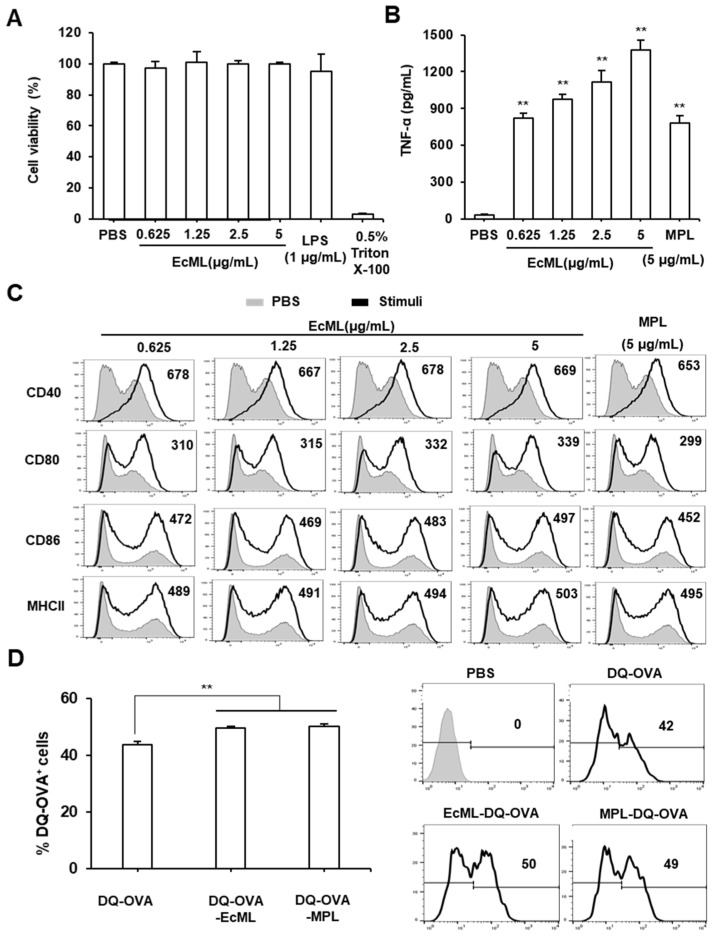
*Escherichia coli*-produced MPL (EcML) significantly enhances the activation and antigen processing of bone marrow-derived dendritic cells (BMDCs). (**A**–**C**) Immature BMDCs were treated with various concentrations of EcML (0.625, 1.25, 2.5, or 5 µg/mL) or 5 µg/mL monophosphoryl lipid A (MPL) for 24 h at 37 °C. (**A**) Cytotoxicity of EcML was evaluated by measuring the cell viability using Trypan blue stain 0.4%. (**B**) Production of TNF-α in the cell culture supernatants was measured by ELISA. (**C**) Cells were stained with fluorescent dye-conjugated anti-CD40, CD80, CD86, and major histocompatibility complex (MHC)-II Abs, and the expression levels of the molecules were then analyzed by flow cytometry. (**D**) Immature BMDCs were incubated with BODIPYL FL dye-conjugated ovalbumin (DQ-OVA), plus either 2.5 µg/mL EcML or 2.5 µg/mL MPL for 5 h at 37 °C. Fluorescent intensity was measured via flow cytometry. The numbers in the histograms indicate the mean fluorescence intensity (MFI) values (**C**) and the percentages of the DQ-OVA^+^ cells (**D**). Data are representative of at least three independent experiments. Statistical differences were analyzed by *t*-test; ** *p* < 0.01.

To examine whether EcML can cause cell cytotoxicity in vitro, we first treated immature BMDCs with various concentrations of EcML (0.625, 1.25, 2.5, or 5 µg/mL), 1 µg/mL LPS, or 0.5% Triton X-100 for 24 h at 37 °C. The cell cytotoxicity was investigated using a Trypan blue assay. As shown in Figure 1A, the treatment of EcML did not affect the cell viability regardless of the tested concentrations.

As DCs play an essential role in the effective induction of adaptive immune responses [24], we next examined the effect of EcML on the activation of DCs in vitro. Immature BMDCs were stimulated with various concentrations of EcML (0.625, 1.25, 2.5, or 5 µg/mL) for 24 h at 37 °C. MPL was used for comparison. The production of TNF-α and the expression of various costimulatory molecules of the stimulated BMDCs were analyzed by ELISA and flow cytometry, respectively. Our results revealed that EcML-treated BMDCs strongly enhanced TNF-α cytokine levels in a dose-dependent manner. The EcML-increased cytokine levels were significantly higher than those enhanced by PBS-treated BMDCs (*p* < 0.01). In particular, BMDCs treated with EcML produced much higher levels of TNF-α cytokine than those treated with MPL at the same concentration (5 µg/mL) (Figure 1B). We also observed that the expression of the costimulatory molecules (CD40, CD80, and CD86) and MHC II molecule were dramatically upregulated on EcML-treated BMDCs compared to those on PBS-treated BMDCs (Figure 1C). The treatment of EcML dose-dependently increased the MFIs of CD80 and MHC-II, while the MFIs of CD86 were increased by treatment with 2.5 μg/mL and 5 μg/mL compared to that with lower concentrations such as 0.625 μg/mL and 1.25 μg/mL. The MFIs of CD40 were independent from the dose of EcML, speculating that the expression of CD40 reached a saturation point even at the lowest concentration (0.625 μg/mL). To investigate antigen processing, we incubated BMDCs with DQ-OVA (a self-quenching dye that emits green fluorescence upon the degradation of OVA) alone or mixed with either 2.5 µg/mL EcML or 2.5 µg/mL MPL. Flow cytometry indicated that the percentages of DQ-OVA^+^ cells significantly increased in BMDCs treated with EcML-DQ-OVA and MPL-DQ-OVA than those treated with DQ-OVA alone (*p* < 0.01) (Figure 1D). Collectively, these results indicate that EcML effectively increases the in vitro activation and antigen processing of DCs.

### 3.2. EcML-Induced DC Activation is Mediated by TLR4

**Figure 2:**
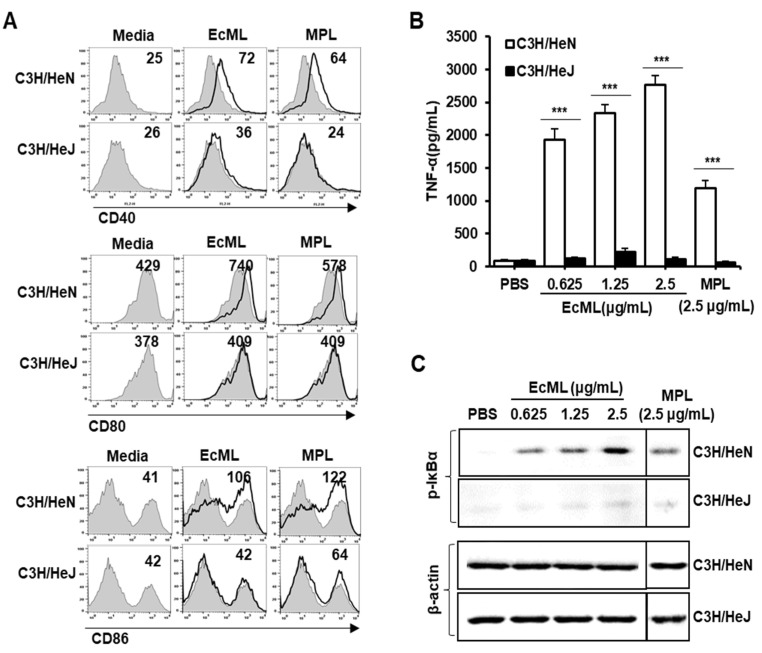
EcML-induced dendritic cell (DC) activation is mediated through Toll-like receptor 4 (TLR4). BMDCs were generated from C3H/HeN and C3H/HeJ mice by culturing in the presence of granulocyte-macrophage colony-stimulating factor and IL-4 for 7 days. (**A**,**B**) The cells were then stimulated with PBS, EcML, or MPL for 24 h at 37 °C. (**A**) Expression levels of CD40, CD80, and CD86 were analyzed by flow cytometry. The numbers in the histograms indicate MFI values. (**B**) The levels of TNF-α cytokine in the culture supernatants were measured by ELISA. (**C**) BMDCs were serum-starved for 3 h and then stimulated with 0.625, 1.25, or 2.5 µg/mL EcML or 2.5 µg/mL MPL for 30 min at 37 °C. Expression levels of phosphorylated-IκBα (p-IκBα) and β-actin in the cell lysates were determined by Western blotting. The data are representative of three independent experiments. Statistical significance was analyzed by *t*-test; *** *p* < 0.001.

Innate immune responses can be activated by pathogen-associated molecular patterns via TLRs, and this plays a critical role in initiating antigen-specific immune responses [25]. A previous study has shown that EcML-induced IFN-ꞵ production was significantly decreased in DCs by pre-treatment with TLR4 neutralizing Ab [26]. Thus, we questioned whether EcML-induced DC activation was mediated by the TLR4 signaling pathway using wild-type C3H/HeN and TLR4-defective C3H/HeJ-generated BMDCs. The BMDCs were exposed to PBS, EcML (2.5 µg/mL), or MPL (2.5 µg/mL) for 24 h at 37 °C. Production of TNF-α cytokine and the expression of costimulatory molecules CD40, CD80, and CD86 were respectively investigated using ELISA and flow cytometry. As shown in Figure 2A, EcML-treated BMDCs from TLR4-defective C3H/HeJ mice expressed lower levels of CD40, CD80, and CD86 than those of EcML-treated BMDCs from wild-type C3H/HeN mice (Figure 2A). Additionally, treatment with EcML resulted in the dose-dependent enhancement of TNF-α cytokine levels from the BMDCs of wild-type mice. By contrast, the cytokine was significantly suppressed in BMDCs from TLR4-defective mice at the same concentration of EcML (*p* < 0.001) (Figure 2B).

As NF-κB activation is a major signaling pathway through which TLR4 induces innate immune responses [27], we examined whether EcML-induced DC activation was mediated through NF-κB activation via TLR4 by evaluating the IκBα phosphorylation in BMDCs generated from wild-type C3H/HeN and TLR4-defective C3H/HeJ mice. Western blot analysis revealed that EcML increased the levels of phosphorylated-IκBα in wild-type BMDCs in a dose-dependent manner. By contrast, the phosphorylated-IκBα levels were low in TLR4-defective BMDCs treated with EcML (Figure 2C). Collectively, these results indicate that the TLR4-mediated NF-κB signaling pathway plays an essential role in EcML-induced DC activation.

### 3.3. EcML Treatment Results in Dose-Dependent Enhancement of Antigen-Specific Humoral and Cellular Immunity

**Figure 3:**
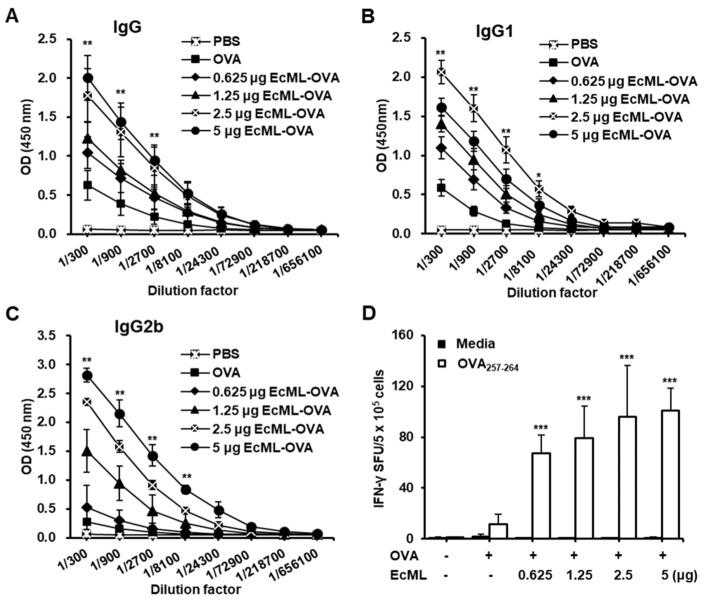
EcML dose-dependently improves OVA-specific humoral and cellular immune responses. C57BL/6 mice (n = 5 per group) were intramuscularly (i.m.) immunized with 10 µg OVA protein alone or mixed with various concentrations of EcML (0.625, 1.25, 2.5, or 5 µg) on days 0, 14, and 28. Sera and spleen were obtained from the immunized mice 14 days after the last immunization. Serum levels of OVA-specific IgG (**A**), IgG1 (**B**), and IgG2b (**C**) were determined by ELISA. (**D**) The number of OVA-specific IFN-γ spot-forming units (SFUs) was determined using the enzyme-linked immunospot (ELISPOT) assay. The data are representative of three independent experiments. Statistically significant differences between EcML–OVA groups versus the OVA group (A–D) were identified by ANOVA/Bonferroni; * *p* < 0.05, ** *p* < 0.01, and *** *p* < 0.001.

An ideal concentration of adjuvants is important to sufficiently induce vaccine efficacy. We immunized C57BL/6 mice with 10 μg OVA protein alone or mixed with 0.625, 1.25, 2.5, or 5 μg EcML (EcML–OVA) on days 0, 14, and 28. Two weeks after the last immunization, the levels of OVA-specific IgG, IgG1, and IgG2b were measured in the sera of the immunized mice using ELISA. Compared to the OVA alone group, OVA-specific IgG levels significantly increased in the groups immunized with OVA plus 1.25, 2.5, or 5 μg EcML when using serum dilution factors of 300, 900, and 2700 (*p* < 0.01) (Figure 3A). There was no statistically significant difference in the IgG level between the 0.625 μg EcML–OVA and OVA alone groups or between 2.5 and 5 μg EcML–OVA groups. Moreover, OVA-specific IgG1 levels significantly increased in all EcML–OVA groups compared to the OVA alone group when using serum dilution factors of 300, 900, 2700, and 8100 (*p* < 0.05) (Figure 3B). OVA-specific IgG2b levels significantly increased in all EcML–OVA groups, except in the 0.625 μg EcML–OVA group, compared to the OVA alone group when using serum dilution factors of 300, 900, 2700, and 8100 (*p* < 0.01) (Figure 3C). While the IgG1 level was the highest in the immunized mice using 2.5 μg EcML, the levels of IgG2b increased and correlated with increasing EcML concentration.

To determine the concentration effects of EcML in enhancing the antigen-specific cellular immune responses, we stimulated the splenocytes of the immunized mice with OVA_257–264_ peptides, which are restricted peptides presented to MHC class I molecules. We observed that the numbers of OVA_257–264_ peptide-specific IFN-γ-producing cells increased significantly in mice vaccinated with OVA plus EcML as compared to mice vaccinated with OVA alone (*p* < 0.001). Nevertheless, no statistically significant differences were observed among the OVA plus EcML groups (Figure 3D). Together, our results suggest that the use of 2.5 μg EcML, combined with antigens, is sufficient to induce both antigen-specific humoral and cellular immune responses in mice.

### 3.4. EcML Significantly Enhances OVA-Specific Cellular Immune Responses Compared to Alum 

**Figure 4:**
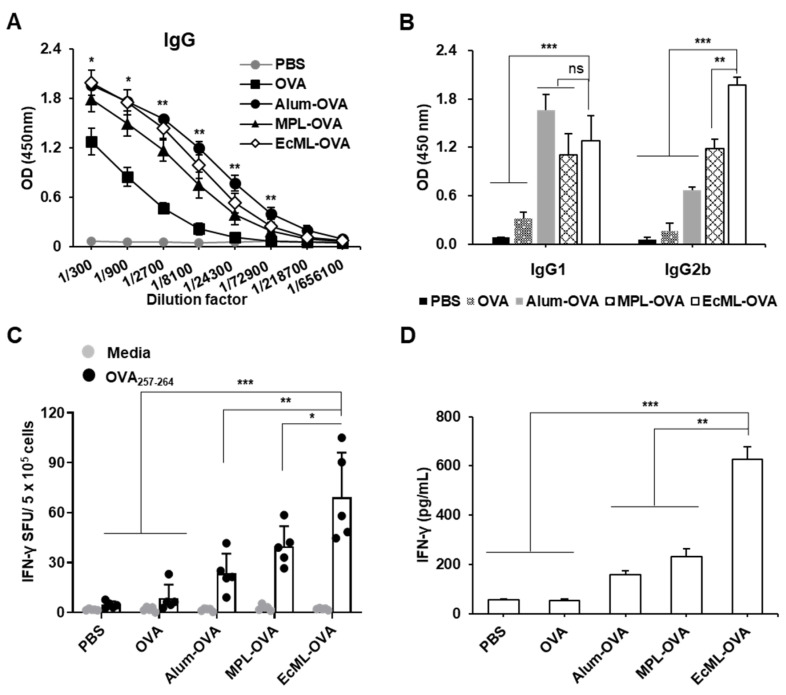
EcML effectively enhances OVA-specific humoral and cellular immune responses compared to alum and MPL. C57BL/6 mice (n = 5 per group) were i.m. immunized with 10 µg OVA protein alone or mixed with 25 µg alum, 2.5 µg MPL, or 2.5 µg EcML. Sera and spleen were obtained from the immunized mice 14 days after the last immunization. (**A**) The serum level of OVA-specific IgG was determined using ELISA. (**B**) Sera were diluted (1:500) in PBS and employed in ELISA to measure the IgG1 and IgG2b levels. (**C**) Splenocytes from the immunized mice were stimulated with OVA_257–264_ peptide (0.5 µg/well) for 3 days, and the number of OVA_257–264_-specific IFN-γ SFUs was assessed using ELISPOT assay. (**D**) Splenocytes from the immunized mice were stimulated with 1 µg/mL OVA protein for 5 days. The levels of IFN-γ in the pooled cell culture supernatants were measured using ELISA. The data are representative of three independent experiments. Statistically significant differences between EcML–OVA group versus other groups (**A**–**D**) were identified by ANOVA/Bonferroni; * *p* < 0.05, ** *p* < 0.01, *** *p* < 0.001, and ns: not significant.

To elucidate the adjuvant effects of EcML, we performed a side-by-side comparison of the adjuvants, including EcML, MPL, and alum. C57BL/6 mice were immunized with 10 μg OVA protein mixed with alum (alum–OVA), MPL (MPL–OVA), or EcML (EcML–OVA) on days 0, 14, and 28. Mice immunized with either OVA protein alone or PBS were used as the negative controls. Two weeks after the final immunization, the levels of OVA-specific Abs in the sera of immunized mice were measured using ELISA. Our results showed that the levels of OVA-specific IgG were significantly higher in the EcML–OVA group than in the OVA alone group when using serum dilution factors of 300, 900, 2700, 8100, and 72,900 (*p* < 0.05) (Figure 4A). The increased IgG levels were shown in the EcML–OVA group compared to MPL–OVA group, but there was no significant difference. Similar levels of OVA-specific IgG were observed between the EcML–OVA and alum–OVA groups, suggesting that EcML may be as powerful as alum in Ab production. Additionally, the level of OVA-specific IgG1 in the serum of mice vaccinated with EcML–OVA was significantly higher than those of the PBS and OVA groups (*p* < 0.001) (Figure 4B). There was no significant difference in the IgG1 level between alum–OVA, MPL–OVA, and the EcML–OVA groups. Notably, the serum level of OVA-specific IgG2b dramatically increased in the EcML–OVA group compared to those of other groups. Furthermore, we performed an ELISPOT assay to compare the adjuvanticity in the enhancement of antigen-specific cellular immunity. As shown in Figure 4C, the number of OVA_257–264_ peptide-specific IFN-γ-secreting cells was significantly higher in the EcML–OVA group (69 ± 27 SFUs) than those in the other groups (5 ± 2 SFUs for PBS, 9 ± 8 SFUs for OVA, 23 ± 18 SFUs for alum–OVA, and 40 ± 12 SFUs for MPL–OVA). We consistently observed that the level of released IFN-γ cytokine was significantly increased in the EcML–OVA group (627 ± 50 pg/mL) compared to those of the other groups (56 ± 4 pg/mL for PBS, 55 ± 5 pg/mL for OVA, 158 ± 18 pg/mL for alum–OVA, and 230 ± 32 pg/mL for MPL–OVA), when using splenocyte culture supernatants upon ex vivo stimulation with OVA protein (Figure 4D). Collectively, these findings indicate that in comparison to both alum and MPL adjuvants, EcML enhances comparable humoral immune responses but more robust cellular immune responses specific to the OVA antigen.

**Figure 5:**
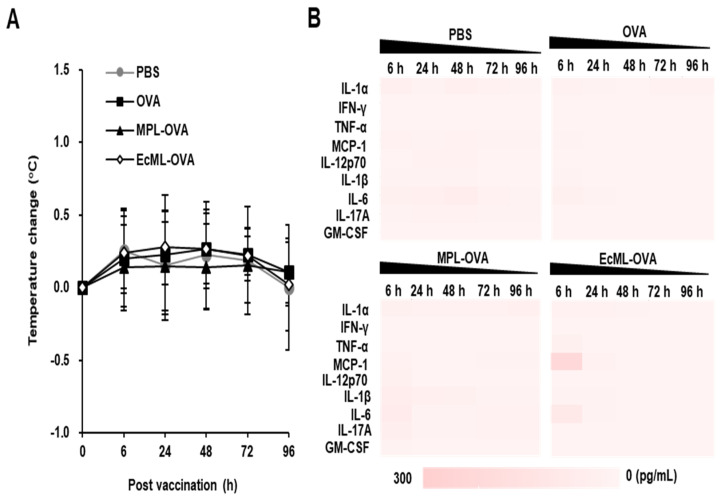
EcML transiently induces systemic inflammatory responses after vaccination. C57BL/6 mice (at least 3 mice per group) were i.m. immunized in tibialis anterior with PBS, 10 µg OVA protein alone or in combination with either 2.5 µg EcML or 2.5 µg MPL. (**A**) The body temperature was measured using a rectal probe. (**B**) Heat map of the inflammatory cytokine levels in the sera of the vaccinated mice as measured using a Legendplex immunoassay kit. The data are representative of three independent experiments.

To investigate whether EcML induces systemic inflammatory responses, we immunized C57BL/6 mice with 10 µg OVA protein alone or in combination with 2.5 µg EcML (EcML–OVA). MPL was used for comparison, and PBS was used as a negative control. Body temperature was measured at 6, 24, 48, 72, and 96 h post-injection. The production of systemic inflammatory cytokines, including IL-1α, IFN-γ, TNF-α, MCP-1, IL-12p70, IL-1β, IL-6, IL-17A, and GM-CSF, was determined in the sera of the immunized mice. As shown in Figure 5A, mice vaccinated with EcML–OVA showed no significant changes in body temperature at different time points after the vaccination compared to the mice immunized with PBS, OVA, or MPL–OVA. Additionally, the levels of MCP-1 and IL-6 were slightly increased in the sera of mice injected with EcML–OVA, but not in other groups at 6 h post-injection. The cytokines returned to baseline levels by 24 h post-immunization (Figure 5B). Collectively, these results indicate that EcML does not affect body temperature and transiently induces systemic inflammatory cytokines after injection.

### 3.5. EcML Enhances the Protective Efficacy of Influenza Vaccine Antigen

**Figure 6:**
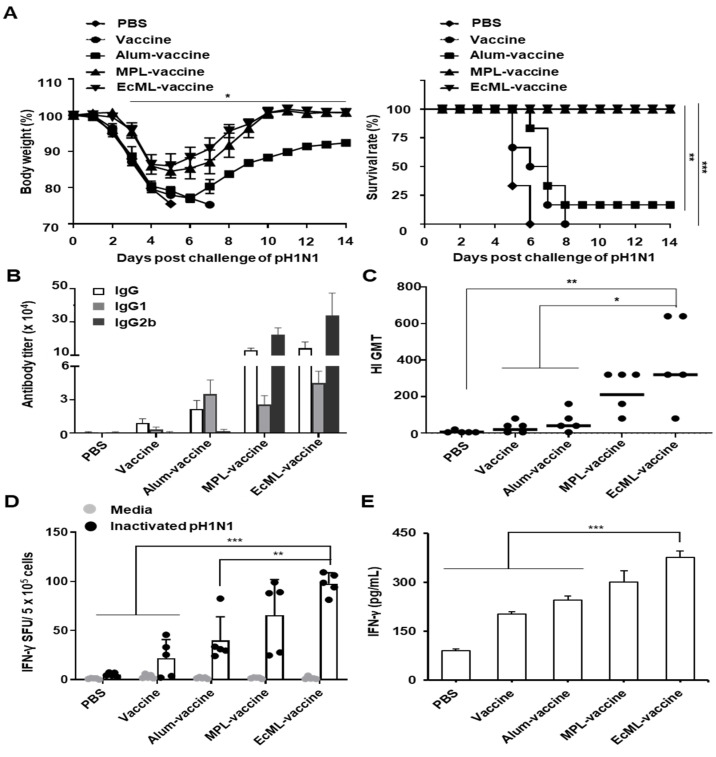
EcML enhances the protective efficacy of the influenza vaccine antigen. C57BL/6 mice (n = 6 per group) were i.m. immunized with 0.05 μg pH1N1 split vaccine antigen combined with 25 μg alum, 2.5 μg EcML, or 2.5 μg MPL on days 0 and 14. (**A**) Two weeks after the last vaccination, the mice were i.n. challenged with 50 LD_50_ pH1N1 influenza virus. Body weight changes and survival rates were monitored up to 14 days post-challenge. Each data point represents the average percentage. (**B**) Sera from the immunized mice were obtained two weeks after the final administration, and the endpoint titers of the influenza vaccine antigen-specific Abs are expressed as the means ± SD. (**C**) Serum HI titers against a pH1N1 virus were measured by HI assay. The lines indicate geometric means, and negative titers were assigned a value of 5 for calculation. (**D**) Splenocytes from the immunized mice were stimulated with UV-inactivated pH1N1 influenza (500 median tissue culture infectious dose (TCID_50_)/well) for 3 days, and the numbers of influenza virus-specific IFN-γ SFUs were then determined using ELISPOT assay. (**E**) Splenocytes from the immunized mice were stimulated with UV-inactivated pH1N1 influenza virus (500 TCID_50_/well) for 5 days. The levels of IFN-γ in the pooled cell culture supernatants were measured using ELISA. The data are representative of three independent experiments. Statistically significant differences were identified by ANOVA/Bonferroni or log-rank test (for survival); * *p* < 0.05, ** *p* < 0.01, and *** *p* < 0.001.

To evaluate whether EcML improves the influenza vaccine efficacy, we examined the effects of EcML on the protection against viral infection resulting from the pH1N1 vaccine. Mice were vaccinated with the pH1N1 split vaccine antigen mixed with alum (alum–vaccine), MPL (MPL–vaccine), or EcML (EcML–vaccine) on days 0 and 14. Vaccine antigen alone or PBS were used as controls. Fourteen days after the last immunization, the immunized mice were i.n. challenged with a lethal dose (50 LD_50_) of the pH1N1 influenza virus. As shown in Figure 6A, mice vaccinated with both EcML–vaccine and MPL–vaccine showed 100% survival without considerable body weight loss during 14 days after the viral challenge. By contrast, the mice vaccinated with alum–vaccine showed severe body weight loss and were partially protected, with 20% survival. Mice immunized with PBS or vaccine alone had 0% survival.

To clarify the adjuvant effects of EcML on the protection against the pH1N1 influenza virus challenge, we examined the influenza-specific humoral and cellular responses including Ab titers, HI titers, CTL activity, and IFN-γ release, using the sera and splenocytes of the vaccinated mice. Our results showed that the influenza vaccine antigen-specific IgG, IgG1, and IgG2b titers were higher in the sera of mice vaccinated with EcML–vaccine than in the sera of mice in the PBS, vaccine, and alum–vaccine groups. There were comparable levels of those Abs between EcML–vaccine and MPL–vaccine groups (Figure 6B). In addition, the HI assay revealed that the HI titers were significantly increased in the sera obtained from the EcML–vaccine group (320 ± 198 geometric mean titer (GMT)) compared to those of sera from PBS (7 ± 6 GMT, *p* < 0.01), vaccine alone (20 ± 26 GMT, *p* < 0.05), and alum–vaccine (40 ± 53 GMT, *p* < 0.05) groups. There was no significant difference in the HI titers of the EcML–vaccine and MPL–vaccine groups (211 ± 101 GMT, *p* = 0.52) (Figure 6C). Moreover, the ELISPOT assay showed that the number of pH1N1 influenza virus-specific IFN-γ secreting cells was significantly higher in the EcML–vaccine group (98 ± 10 SFUs) than those in PBS (5 ± 2 SFUs, *p* < 0.001), vaccine alone (21 ± 19 SFUs, *p* < 0.001), and alum–vaccine (40 ± 21 SFUs, *p* < 0.01) groups. No significant difference in the cell number was observed between the EcML–vaccine and the MPL–vaccine (66 ± 33 SFUs, *p* = 0.3) groups (Figure 6D). Additionally, the level of IFN-γ secreted in the splenocyte culture supernatant of mice vaccinated with EcML–vaccine (375 ± 20 pg/mL) was also significantly higher than that of the PBS (90 ± 6 pg/mL), vaccine (202 ± 7 pg/mL), and the alum–vaccine (246 ± 12 pg/mL) groups (*p* < 0.001). There was no significant difference in the cytokine levels of the EcML–vaccine and MPL–vaccine (301 ± 33 pg/mL, *p* < 0.06) groups (Figure 6E). Collectively, our results suggest that EcML improves the protective efficacy of the pH1N1 influenza vaccine antigen by enhancing influenza-specific humoral and cellular immune responses.

## 4. Discussion

Despite the availability of annual vaccinations, influenza remains a significant cause of human infectious disease and, thus, there is a push to improve vaccine effectiveness. An adjuvant capable of enhancing vaccine immunogenicity without compromising safety would be beneficial to improving influenza vaccines. However, the main influenza vaccine adjuvants, including alum, MF59, and AS03, have limitations such as their weak induction of cellular immune responses and safety issues [4,5,6,7,8] that need to be addressed. MPL has been used as an adjuvant in several human vaccines [19] and has reported increasing influenza vaccine efficacy [28]. However, its disadvantages, including the difficult manufacturing processes and prohibitive cost, have precluded its use as an easy-to-use and cost-effective adjuvant. Thus, an alternative component could be interesting and, in fact, *E. coli*-produced MPL (named EcML) has recently been produced by direct extraction from an engineered *E. coli* [20]. The *E. coli* strain is a sustainable source and can be easily grown at a very low cost and EcML is directly extracted from the bioengineered *E. coli* without hydrolysis, indicating that the manufacture of EcML is more cost-effective than that of MPL [20]. Here, we revealed that EcML might be safe to use due to no in vitro cell cytotoxicity, unchanged body temperature, and transient systemic inflammatory responses after vaccination in mice. In addition, EcML robustly activated DCs via the TLR4-mediated NF-κB signaling pathway and increased the antigen processing of cells in vitro. Moreover, EcML enhanced antigen-specific humoral and cellular immune responses. Ultimately, EcML effectively improved the protective immunity of the pH1N1 influenza vaccine antigen. 

DCs play a major role in innate immunity and serve as a significant link between innate and adaptive immunity [29]. Thus, targeting antigens to DCs is a crucial strategy in vaccine development. Previously, it was also reported that EcML dose-dependently enhanced the BMDC phagocytic activity against B16F10 melanoma cells [26]. In this study, we showed that EcML triggered DC activation, including the high production of inflammatory cytokines and the upregulation of the expression of costimulatory and MHC-II molecules. The upregulation of these molecules has been previously seen when using MF59 adjuvants [30]. In addition, we observed that EcML-treated BMDCs had much higher levels of TNF-α and IL-6 (Figure S1) than MPL-treated BMDCs. This result suggests that EcML might be better than MPL in enhancing innate immune responses by DCs. TLR4 agonists can activate innate immune responses and consequently augment adaptive immune responses by enhancing Th1-biased responses [31,32]. A previous study has demonstrated that MPL stimulated innate immunity via TLR4 [33]. In this study, we also observed that EcML activated BMDCs via TLR4-mediated NF-κB signaling, conferring the suppressed production of inflammatory cytokines and the low expression of costimulatory molecules (CD40, CD80, and CD86) in TLR4-defective BMDCs. These results are consistent with the previous data showing that EcML-induced IFN-ꞵ production was significantly decreased in DCs by a TLR4-neutralizing antibody [26]. The antigen processing by antigen-presenting cells is an essential step in presenting antigens to T cells and initiating antigen-specific adaptive immunity [34]. We showed that EcML more efficiently facilitated the degradation of the DQ-OVA antigen, indicating that EcML enhances antigen processing, thereby enhancing adaptive immune responses.

The safety of a vaccine adjuvant is paramount as vaccines are given prophylactically to healthy individuals. A previous study reported that Lipid A, a toxic domain of LPS, was not detected after the purification of EcML, suggesting that EcML is not contaminated with LPS [20]. EcML has also been demonstrated to neither significantly increase the spleen weight nor decrease mice body weight after intravenous injection [26], indicating that EcML may be appropriate for systematic administration. In this study, we further examined the safety of EcML by measuring the cell viability after in vitro treatment and changes in body temperature and systemic inflammatory responses after i.m. immunization in mice. We observed that EcML treatment did not result in any in vitro cytotoxic effects on antigen-presenting cells, including BMDCs and macrophage cell line RAW 264.7 cells (Figure S2). Previous studies have reported that children vaccinated with the MF59-adjuvanted influenza vaccine showed adverse reactions, including redness and swelling at the injection sites [35,36]. We did not observe any such adverse effects in mice after i.m. vaccination with EcML. Mice vaccinated with LPS (an unsafe adjuvant) showed hypothermia which lowered the survival rate after challenge [37]. In this study, there was no significantly different change in body temperature in the mice vaccinated using EcML. While inflammatory responses are a crucial defense mechanism against viral infection, excessive and persistent inflammation can be detrimental [38]. Previous studies demonstrated that side effects are mediated through the systemic distribution of TNF-α and IL-6 [39,40]. Here, we reported that these cytokines subsided to basal levels in the sera of EcML–OVA-vaccinated mice at 24 h post-injection. Our results indicate that EcML induces transient inflammatory responses after vaccination, suggesting that EcML might be safe to use.

A novel vaccine adjuvant could have the ability to improve both antigen-specific humoral and cellular immunity [41]. Consistent with a previous study showing that EcML enhanced OVA-specific antibody response in a BALB/c mouse model [20], our results show that EcML improved OVA-specific Ab production. Moreover, we observed enhanced OVA-specific cellular immune responses by EcML after vaccination using C57BL/6 mice. In particular, EcML increased the number of MHC class I-restricted OVA_257–264_ peptide-specific IFN-γ-producing T cells and the levels of IFN-γ release, indicating that EcML robustly enhances antigen-specific CTL activity. Additionally, we observed that EcML was better than MPL in improving OVA-specific humoral and cellular immune responses. We speculated that the adjuvanticity of EcML could be with the reason behind the enhanced levels of inflammatory responses observed for EcML compared to MPL. Currently, alum and MF59 are mainly used in influenza vaccines [4], but these adjuvants primarily induce responses toward the Th2-biased immunity [4,42] and may not be sufficiently effective in the protection against influenza virus infections. We then evaluated the efficacy of EcML as an adjuvant for the influenza vaccine, in comparison to alum. We found that EcML robustly enhanced not only humoral immune responses, including Ab production and HI titers, but also cellular immune responses, including CTL activity and IFN-γ release. Notably, EcML-induced adaptive immune responses were higher than those induced by alum. Consequently, EcML fully protected mice (100% survival rate), while alum only partially protected mice (20% survival rate) against pH1N1 influenza virus infection.

MPL has been reported to enhance the protective efficacy of the influenza vaccine [28]. We observed that EcML provided similarly protective efficacy with MPL, indicating that EcML was as good as MPL in improving the protective immunity of the pH1N1 vaccine. The induction of the cross-protective immunity of the pH1N1 vaccine is beneficial for the protection against different influenza virus subtypes. We observed that the EcML-vaccine group had an enhanced cross-reactive IFN-γ response against H1N1 (A/Puerto Rico/8/34) and reassortant H3N2 (HA and neuraminidase of A/Hong Kong/1/1968 and internal genes of A/Puerto Rico/8/34) by enhancing the release of H1N1- and H3N2- specific-IFN-γ cytokines into the splenocyte culture supernatant after ex vivo stimulation (Figure S3). Further studies should be performed to clarify this effect of EcML. A combination of different adjuvants in specific formulations can result in the complementary and even synergistic enhancement of immune responses to specific antigens. We also previously reported that a complex of poly-γ-glutamic acid and alum strongly induced the cross-protective efficacy of the pH1N1 vaccine, as compared to each component alone [13]. The AS04 adjuvant composed of MPL and alum has been used in licensed vaccines for human papillomavirus and hepatitis B virus [43,44]. Thus, the combination of EcML with other adjuvants, such as alum, should be further studied to broaden the use of EcML in vaccines against other infectious diseases.

## 5. Conclusions

In an attempt to seek a potent vaccine adjuvant capable of enhancing both cellular and humoral immune responses, we assessed EcML—an MPL extracted from the engineered *E. coli* [20]. EcML was shown to be safe to use, with no in vitro cell cytotoxicity and transient systemic inflammatory responses observed in mice after vaccination. EcML robustly induced innate immune responses, including the activation and antigen processing of DCs, thereby resulting in enhanced antigen-specific humoral and cellular immunities. Importantly, EcML drastically increased the protective efficacy of the pH1N1 vaccine against influenza virus infection by enhancing Ab production, HI titers, CTL activity, and IFN-γ release. We conclude that EcML is a promising adjuvant that may be capable of providing protection from influenza virus infections.

## Supplementary Materials

The following are available online at https://www.mdpi.com/2076-393X/8/2/306/s1, Figure S1: EcML enhances IL-6 cytokine levels in BMDCs in vitro, Figure S2: In vitro cytotoxicity of EcML, Figure S3: EcML enhances cross-reactive IFN-γ responses after vaccination.
