# Supplementary material for: E. coli-Produced Monophosphoryl Lipid a Significantly Enhances Protective Immunity of Pandemic H1N1 Vaccine
Source: Vaccines (Basel). 2020 Jun 16;8(2):306. doi: 10.3390/vaccines8020306 (PMC7350214; doi:10.3390/vaccines8020306)
Supplement: Supplementary file 1 [file vaccines-08-00306-s001.pdf]

vaccines

MDPI

*Supplementary Material*

# *E. coli*-produced Monophosphoryl Lipid A Significantly Enhances Protective Immunity of Pandemic H1N1 Vaccine

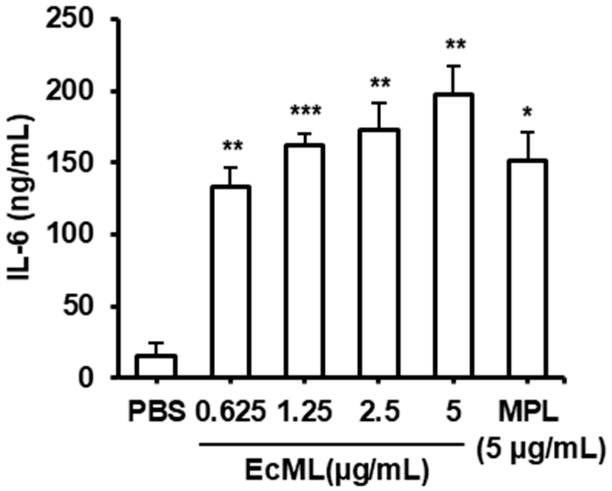


**Figure S1**. EcML enhances IL-6 cytokine levels in BMDCs in vitro. Immature BMDCs were treated with various concentrations of EcML (0.625, 1.25, 2.5, or 5 μg/mL) and 5 μg/mL MPL for 24 h at 37 °C. Levels of IL-6 in the culture supernatants were measured by ELISA. The data are representative of at least three independent experiments. Statistic difference was analyzed by *t*-test; * $P < 0.05$, ** $P < 0.01$, *** $P < 0.001$

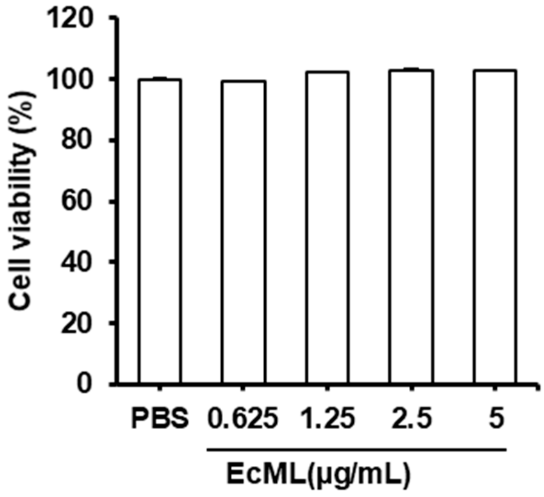


**Figure S2**. In vitro cytotoxicity of EcML. RAW 264.7 cells were treated with various concentrations of EcML (0.625, 1.25, 2.5, or 5 μg/mL) for 24 h at 37 °C. (A) The cytotoxicity of EcML was evaluated by measuring the cell viability of the treated RAW 264.7 cells using the CytoX™ cell viability assay kit. The data are representative of at least three independent experiments.

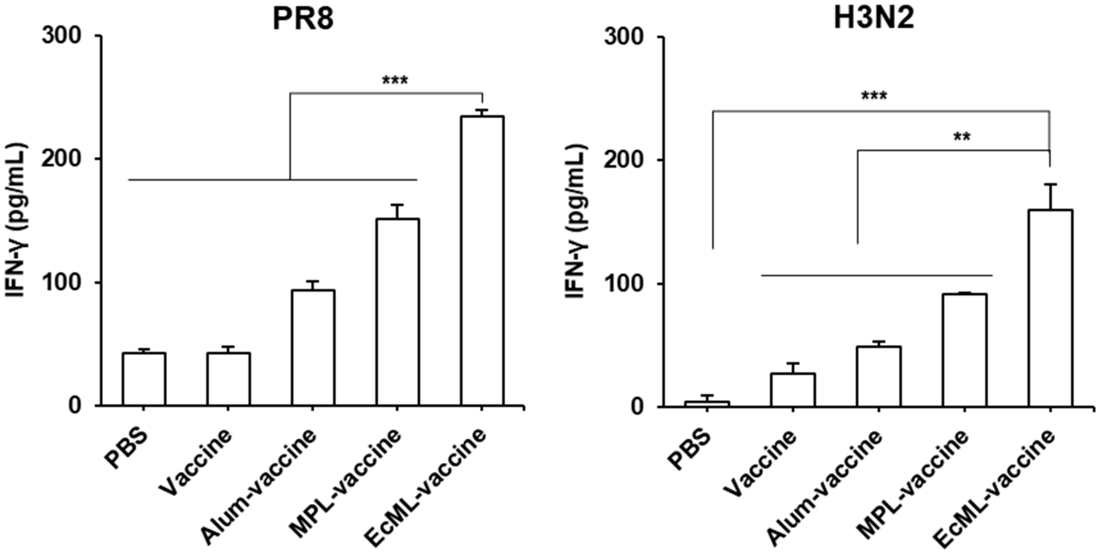


**Figure S3**. EcML enhances cross-reactive IFN-γ responses after vaccination. C57BL/6 mice (n = 6 per group) were i.m. immunized with the 0.05 μg pH1N1 split vaccine antigen combined with 25 μg alum, 2.5 μg EcML, or 2.5 μg MPL on days 0 and 14. Splenocytes were collected from the immunized mice two weeks after the last vaccination and were then stimulated with 500 $TCID_{50}$/well of UV-inactivated influenza H1N1 (A/Puerto Rico/8/34) or reassortant H3N2 (HA and neuraminidase of A/Hong Kong/1/1968 and internal genes of A/Puerto Rico/8/34) viruses for 5 days. The levels of IFN-γ in the culture supernatants of pooled samples were measured using ELISA. The data are representative of three independent experiments with similar results. Statistically significant differences were identified by ANOVA/Bonferroni; $^{**}P < 0.01$, $^{***}P < 0.001$.
